# DAXX promotes SUMOylation of chromatin-trapped DNMT1

**DOI:** 10.1093/jb/mvag011

**Published:** 2026-02-13

**Authors:** Shota Tanimoto, Teh-Wei Wang, Tomohiro Yabushita, Yoshie Chiba, Chieko Konishi, Akinori Endo, Yasushi Saeki, Susumu Goyama, Makoto Nakanishi, Atsuya Nishiyama

**Affiliations:** Division of Cancer Cell Biology, The Institute of Medical Science, The University of Tokyo, 4-6-1 Shirokanedai, Minato-ku, Tokyo 108-8639, Japan; Division of Cancer Cell Biology, The Institute of Medical Science, The University of Tokyo, 4-6-1 Shirokanedai, Minato-ku, Tokyo 108-8639, Japan; Project Division of Generative AI Utilization Aging Cells, The Institute of Medical Science, The University of Tokyo, 4-6-1 Shirokanedai, Minato-ku, Tokyo 108-8639, Japan; Division of Cellular Therapy, The Institute of Medical Science, The University of Tokyo, 4-6-1 Shirokanedai, Minato-ku, Tokyo 108-8639, Japan; Division of Cancer Cell Biology, The Institute of Medical Science, The University of Tokyo, 4-6-1 Shirokanedai, Minato-ku, Tokyo 108-8639, Japan; Division of Cancer Cell Biology, The Institute of Medical Science, The University of Tokyo, 4-6-1 Shirokanedai, Minato-ku, Tokyo 108-8639, Japan; Laboratory of Protein Metabolism, Tokyo Metropolitan Institute of Medical Science, 2-1-6 kamikitazawa, setagaya-ku, Tokyo 156-8506, Japan; Laboratory of Protein Metabolism, Tokyo Metropolitan Institute of Medical Science, 2-1-6 kamikitazawa, setagaya-ku, Tokyo 156-8506, Japan; Division of Protein Metabolism, The Institute of Medical Science, The University of Tokyo, 4-6-1 Shirokanedai, Minato-ku, Tokyo 108-8639, Japan; Division of Molecular Oncology, Department of Computational Biology and Medical Sciences, Graduate School of Frontier Sciences, The University of Tokyo, 4-6-1 Shirokanedai, Minato-ku, Tokyo 108-8639, Japan; Division of Cancer Cell Biology, The Institute of Medical Science, The University of Tokyo, 4-6-1 Shirokanedai, Minato-ku, Tokyo 108-8639, Japan; Division of Cancer Cell Biology, The Institute of Medical Science, The University of Tokyo, 4-6-1 Shirokanedai, Minato-ku, Tokyo 108-8639, Japan

**Keywords:** DAXX, DNA methylation, DNMT1, SUMO, *Xenopus* egg extracts

## Abstract

The cytosine analogue 5-aza-2′-deoxycytidine (decitabine; DAC) covalently inhibits DNA methyltransferase 1 (DNMT1), the maintenance DNA methyltransferase, and induces SUMOylation of DNA-trapped DNMT1, promoting proteasomal degradation. However, the regulation of DNMT1 SUMOylation and its biological impact remain unclear. Here, using interphase *Xenopus* egg extracts that reconstitute maintenance DNA methylation *in vitro*, we identify death domain-associated protein (DAXX) as a regulator of SUMOylation on chromatin-retained, inactive DNMT1. First, adding 5-aza-dCTP, the triphosphate form of decitabine, induced DNMT1 accumulation on chromatin and robust SUMO2/3 conjugation. Chromatin mass spectrometry (CHROMASS) revealed DAXX enrichment on 5-aza-dCTP-treated chromatin in a SUMO-dependent manner, and DAXX depletion suppressed 5-aza-dCTP-induced DNMT1 SUMOylation. Furthermore, GSK-3484862, a non-covalent DNMT1 inhibitor, drove DNMT1 accumulation on chromatin and triggered DAXX-dependent DNMT1 SUMOylation. We also demonstrated that DAXX loss increased decitabine sensitivity in the myeloid leukaemia (AML) cell line THP-1. Together, these findings show that DAXX promotes SUMOylation of DNMT1 trapped on DNA under both covalent and non-covalent trapping conditions.

## Graphical Abstract

**Graphical Abstract:**
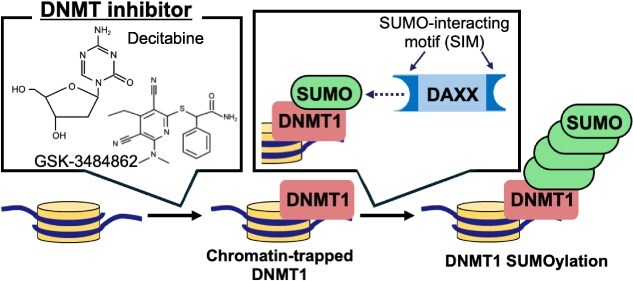

## Abbreviations

AML: acute myeloid leukaemia
CHROMASS: chromatin mass spectrometry
DAC: decitabine
DAXX: death domain-associated protein
DNMT1: DNA methyltransferase 1
DPC: DNA-protein crosslink
HMA: hypomethylating agent
MDS: myelodysplastic syndromes
PAF15: PCNA-associated protein 15
PML: promyelocytic leukaemia
SAM: S-adenosyl-L-methionine
SIM: SUMO-interacting motif
STUbL: SUMO-targeted ubiquitin ligase
SUMO: small ubiquitin-like modifier
UBC9-DN: dominant-negative UBC9

DNA methylation is a key epigenetic mark and plays an essential role in regulating gene expression, transposon silencing, genome stability and genomic imprinting, thereby defining cellular identity (*1*, *2*). In vertebrates, cytosines within CpG dinucleotides are symmetrically methylated at position C5 on both DNA strands. DNA methylation patterns are established during developmental and differentiation processes, and their precise inheritance is essential for maintaining cellular identity (*3*, *4*). DNA methyltransferase 1 (DNMT1) is the key enzyme for maintenance of DNA methylation. DNMT1 transfers a methyl group from S-adenosyl-L-methionine (SAM) to cytosine at hemimethylated CpG sites that arise immediately after replication, thereby restoring a fully methylated state (*5*, *6*). Therefore, DNMT1 deficiency or impaired activity causes genome-wide aberrant DNA methylation, leading to embryonic lethality and various human diseases (*7*, *8*). In cancers, regulatory regions of tumour suppressor and lineage-determining genes, most notably promoter CpG islands and adjacent shores, are frequently hypermethylated, resulting in their reduced expression (*9*). DNMT1 overexpression has been associated with the maintenance of these aberrant patterns in daughter cells (*10*). Conversely, pharmacological inhibition of DNMT1 provides a rationale for hypomethylating agents (HMAs) that target DNMT1 activity (*10*, *11*).

The nucleoside analogue decitabine (5-aza-2′-deoxycytidine; DAC) is an HMA used to treat myelodysplastic syndromes (MDS) and acute myeloid leukaemia (AML) (*12*). After cellular uptake, DAC is phosphorylated to 5-aza-dCTP, which is incorporated during DNA replication and, due to the nitrogen at position C5, blocks methyl transfer, leading to an irreversible DNMT1-DNA complex (*13*). Crosslinked DNMT1 is targeted for proteasomal degradation, leading to DNMT1 depletion and hypomethylation of tumour suppressor genes, which restores their expression *(**14–17*). Recent studies further show that covalent 5-aza-dC-DNMT1 adducts trigger mitotic defects and cell death (*18*). Collectively, these findings indicate that DAC’s antitumour effects derive not only from hypomethylation but also from DNA-protein crosslinks (DPCs) that are highly cytotoxic.

DPC is a kind of bulky DNA damage induced by various endogenous or exogenous factors (*19–21*). During repair of DPC, post-translational modifications such as SUMOylation, mediated by the small ubiquitin-like modifier (SUMO), and ubiquitination play critical roles. DPC repair occurs through both replication-dependent and replication-independent pathways, with SUMOylation of crosslinked proteins being particularly important in the replication-independent pathway (*22*, *23*). Recent studies have revealed that crosslinked DNMT1 undergoes modification with SUMO2/3, a member of the SUMO family capable of forming poly-SUMO chains, and is subsequently ubiquitinated by SUMO-targeted ubiquitin ligases (STUbLs) such as RNF4 and TOPORS (*24–26*). In our previous findings, although TOPORS contains both ubiquitin and SUMO E3 ligase activities, we found that TOPORS knockout only diminished the polyubiquitination of crosslinked DNMT1 but not SUMOylation, which needs further elucidation (*26*). Furthermore, in haematopoietic malignancies, combined treatment with decitabine and the SUMO inhibitor, TAK981, has been reported to delay DNMT1 degradation and enhance chemotherapeutic effects (*26*, *27*). The ubiquitinated DNMT1 is then either (*1*) extracted from chromatin by VCP/p97 (Cdc48) and degraded by the 26S proteasome or (*2*) proteolytically cleaved by the SprT family metalloprotease SPRTN in humans (*14*, *22*, *28*, *29*). However, the molecular mechanisms regulating the formation of poly-SUMO chains during 5-aza-dC-DNMT1 crosslinking remain largely unknown.

In this study, we utilized a cell-free system derived from *Xenopus* egg extracts to reconstruct the maintenance DNA methylation process during DNA replication (*30–32*). Through this system, we demonstrated that the addition of the decitabine metabolite 5-aza-dCTP to the egg extract reproduces DNMT1 accumulation on chromatin and its SUMOylation, consistent with previous observations (*14*, *22*). Furthermore, we identified DAXX as a key regulator of SUMOylation of crosslinked DNMT1, which accumulates on chromatin in a DNMT1- and SUMOylation-dependent manner, thereby promoting formation of poly-SUMO chains.

## Results

### 5-Aza-dCTP traps DNMT1 and induces its SUMOylation in *Xenopus* egg extracts

**Fig. 1:**
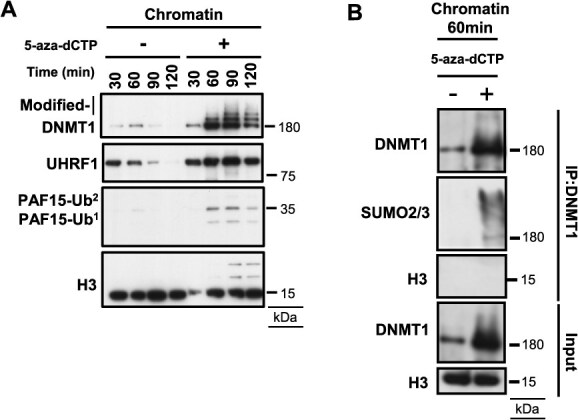
***Xenopus* egg extract recapitulates 5-aza-dCTP-induced SUMOylated DNMT1 accumulation on chromatin.** (A) *Xenopus* sperm nuclei were incubated in egg extracts supplemented with or without 50 μM 5-aza-dCTP for indicated periods. Isolated chromatins were analyzed by immunoblotting with the indicated antibodies. (B) Immunoblotting results using indicated antibodies following DNMT1 immunoprecipitation of chromatin treated or untreated with 5-aza-dCTP for 60 min. The input results were shown in the lower panels.

*Xenopus* egg extracts provide a powerful biochemical cell-free system that recapitulates DNA replication-associated chromatin events, including maintenance DNA methylation (*30*, *33*). In this system, the addition of sperm chromatin to egg extracts leads to the assembly of functional nuclei that undergo a single round of DNA replication. We previously showed that DNMT1 chromatin recruitment and enzymatic activation during DNA replication require UHRF1-mediated ubiquitination of histone H3 and PCNA-associated protein 15 (PAF15) in *Xenopus* egg extracts (*30–32*, *34*, *35*). To investigate how 5-aza-dCTP impacts DNMT1 behaviour in this system, we examined the effect of adding 5-aza-dCTP to *Xenopus* egg extracts. As previously reported, in the control reaction, DNMT1 was transiently recruited to chromatin during DNA replication, coinciding with UHRF1-dependent ubiquitination and PAF15 loading. In contrast, in the presence of 5-aza-dCTP, DNMT1 chromatin association was markedly enhanced and became persistent. Moreover, 5-aza-dCTP prevented the dissociation of UHRF1 and ubiquitinated PAF15 from chromatin (Fig. 1A). These observations suggested that hemimethylated DNA was not converted to fully methylated DNA and that DNMT1 methyltransferase activity was inhibited by 5-aza-dCTP in the egg extracts. Notably, upon 5-aza-dCTP addition, chromatin-bound DNMT1 appeared as a ladder of slower-migrating species (Fig. 1A). Immunoprecipitation of DNMT1 from chromatin fraction revealed that 5-aza-dCTP treatment led to prominent DNMT1 SUMOylation involving SUMO2/3 (Fig. 1B). Together, these data indicate that 5-aza-dCTP-induced DNMT1 inactivation, accompanied by DNMT1 SUMOylation, can be recapitulated in *Xenopus* egg extracts.

### DAXX is recruited to DNMT1-DNA crosslinks in a SIM-dependent manner to promote DNMT1 SUMOylation

**Fig. 2:**
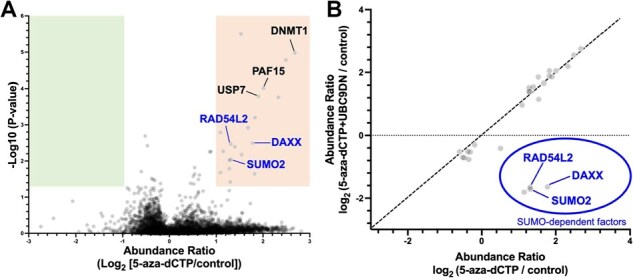
**DAXX accumulates on DNMT1-trapped chromatin in a SUMO-dependent manner identified by CHROMASS.** (A) Volcano plot showing chromatin mass spectrometry analysis of protein recruitment to chromatins treated with or without 5-aza-dCTP (*n* = 3 technical replicates). Proteins highlighted in blue were identified in the comparison between 5-aza-dCTP+UBC9-DN and control groups and represent factors whose accumulation on chromatin is associated with SUMOylation. (B) Scatter plot showing protein abundance ratios derived from indicated comparisons in the axis titles.

Next, to identify regulators of DNMT1 SUMOylation at DNMT1-DNA crosslinks, we applied chromatin mass spectrometry (CHROMASS) (*36*). Replicated sperm chromatin was isolated by centrifugation and analyzed by LC–MS (*n* = 3) under three conditions: mock control; 5-aza-dCTP treatment; and 5-aza-dCTP in combination with dominant-negative UBC9 (UBC9-DN) (*37*) to inhibit the SUMO pathway. Among 6803 identified proteins, only 20 exhibited significant changes between the mock control and 5-aza-dCTP-treated samples (Fig. 2A). As expected, 5-aza-dCTP induced robust enrichment of DNMT1, USP7 and PAF15. Importantly, SUMO2, RAD54L2 (also known as ARIP4) and death domain-associated protein (DAXX) were detected on 5-aza-dCTP-treated chromatin in a SUMO-dependent manner (Fig. 2A and B).

**Fig. 3:**
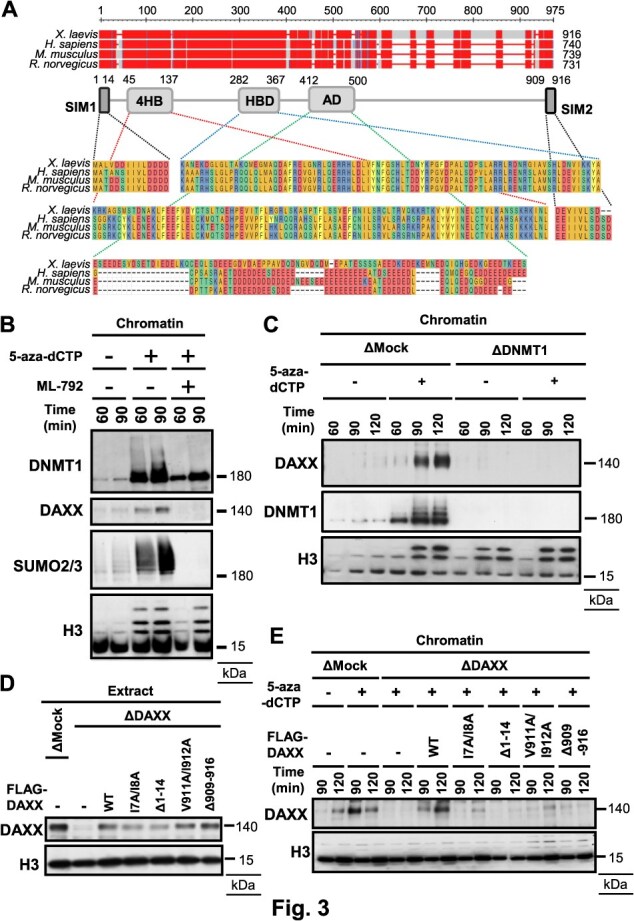
**DAXX interacts with SUMOylated DNMT1 on chromatin through SIMs.** (A) The multiple sequence alignment of DAXX from different indicated species. DAXX contains SUMO-interacting motifs (SIMs), four-helix bundle (4HB), histone binding domain (HBD) and acidic residue-rich domain (ad). (B) *Xenopus* sperm nuclei were incubated in egg extracts supplemented with buffer, 5-aza-dCTP or 5-aza-dCTP and SUMO inhibitor ML-792. Isolated chromatin was analyzed by immunoblotting with indicated antibodies. (C) *Xenopus* sperm nuclei were incubated in mock-depleted or DNMT1-depleted extracts in the presence of buffer or 5-aza-dCTP. Isolated chromatin was analyzed by immunoblotting with indicated antibodies. (D) 3× FLAG-tagged xDAXX WT, 3× FLAG-tagged xDAXX SIM1 mutants (I7AI8A point mutant or 1–14 deletion mutant) or 3× FLAG-tagged xDAXX SIM2 mutants (V911A/I912A point mutant or 909–916 deletion mutant) were added to DAXX-depleted extracts. (E) *Xenopus* sperm nuclei were incubated in mock-depleted or DAXX-depleted extracts supplemented with xDAXX WT, xDAXX SIM1 mutants or xDAXX SIM2 mutants in the presence of buffer or 5-aza-dCTP.

DAXX is a multifunctional scaffold protein that interacts with the SUMO-conjugating enzyme UBC9, SUMO2/3 and promyelocytic leukaemia (PML) proteins through its two SUMO-interacting motifs (SIMs) located near the N- and C-termini (*38–42*). Besides, DAXX also recognizes SUMOylated proteins such as MORC3 or PRC1, then promotes histone H3.3 incorporation during histone remodelling (*41*, *42*). Although *Xenopus* DAXX (xDAXX) contains approximately 170 additional amino acids, the 4-helix bundle (4HB), histone-binding domain (HBD) and both SIMs remain highly conserved at the protein sequence level (Fig. 3A). To analyze chromatin binding of xDAXX during DNA replication, we generated an antibody against a bacterially expressed xDAXX fragment containing amino acids 362–916. While 5-aza-dCTP addition induced DAXX accumulation on chromatin, SUMO E1 inhibition with ML-792 or DNMT1 depletion from egg extracts abolished this accumulation (Fig. 3B and C), indicating that DNMT1 SUMOylation is required for xDAXX recruitment to chromatin upon 5-aza-dCTP treatment.

To determine whether SUMO binding is necessary for xDAXX recruitment, we depleted endogenous xDAXX using anti-xDAXX antibody and supplemented the extracts with recombinant wild-type xDAXX, ΔSIM1, ΔSIM2 or SIM hydrophobic-core mutants (I7A/I8A or V911A/I912A), and then assessed chromatin loading (Fig. 3D and E). Compared with wild-type, deletion of SIM1 or SIM2, or hydrophobic-core mutations within these SIMs, reduced xDAXX chromatin recruitment in 5-aza-dCTP-treated extracts (Fig. 3D and E). These results suggest that xDAXX accumulates at DNMT1-DNA crosslinks via its SUMO binding.

**Fig. 4:**
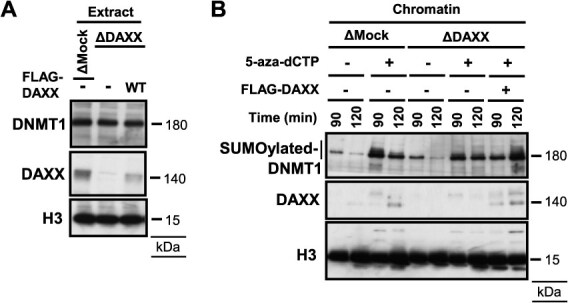
**DAXX promotes SUMOylation of chromatin-trapped DNMT1.** (A) Immunoblotting results of egg extracts using indicated antibodies. 3× FLAG-tagged xDAXX WT was added to xDAXX-depleted egg extracts. (B) Chromatin fractions were isolated from mock-depleted or xDAXX-depleted egg extracts treated or untreated with 5-aza-dCTP for the indicated times, with or without adding 3× FLAG-tagged xDAXX. DNMT1 SUMOylation was assessed by immunoblotting using anti-DNMT1 antibodies.

To define the regulatory role of xDAXX in DNMT1 SUMOylation, we analyzed DNMT1 modification in xDAXX-depleted extracts. During the unperturbed S phase, xDAXX depletion had only a minor effect on DNMT1 chromatin recruitment (Fig. 4A and B). Meanwhile, xDAXX depletion suppressed DNMT1 SUMOylation induced by 5-aza-dCTP, and re-addition of recombinant FLAG-xDAXX restored DNMT1 SUMOylation (Fig. 4B). These findings support a model in which xDAXX is recruited to SUMOylated DNMT1 at DNMT1-DNA crosslinks via its SIMs and, once bound, promotes further DNMT1 SUMOylation.

### Non-covalent DNMT1 inhibitor GSK-3484862 induces DAXX-dependent DNMT1 SUMOylation

**Fig. 5:**
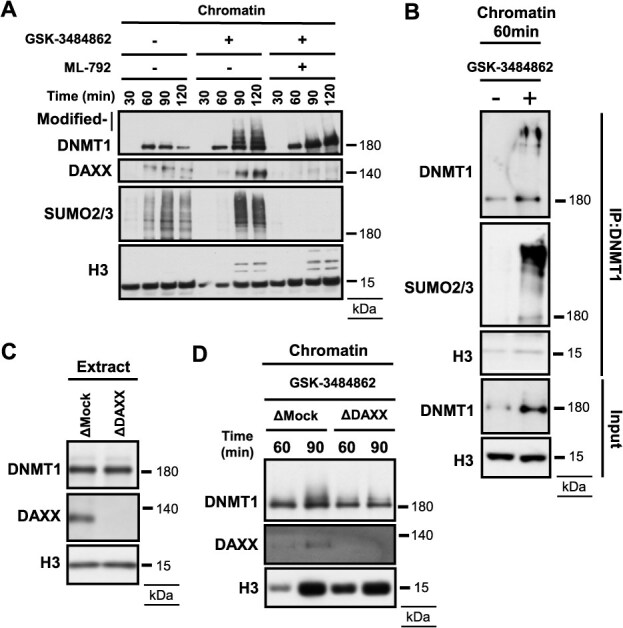
**Non-covalent DNMT1 inhibitor GSK-3484862 induces DAXX-regulated DNMT1 SUMOylation.** (A) *Xenopus* sperm nuclei were incubated in egg extracts supplemented with DMSO, GSK-3484862 or GSK-3484862 and ML-792. Isolated chromatin was analyzed by immunoblotting with indicated antibodies. (B) Immunoblotting results using indicated antibodies following DNMT1 immunoprecipitation of chromatin treated with or without GSK-3484862 for 60 min. The input results are shown in the lower panels. (C) Immunoblotting results of mock-depleted or DAXX-depleted egg extracts using indicated antibodies. (D) *Xenopus* sperm nuclei were incubated in mock-depleted or DAXX-depleted extracts treated with GSK-3484862 for 60 or 90 min. Isolated chromatin was analyzed by immunoblotting with indicated antibodies.

Previous reports have shown that formation of DNMT1-DNA crosslinks is critical for DNMT1 SUMOylation. However, recent reports have demonstrated that PARP inhibitors promote PARP1 SUMOylation, which is associated with non-covalent trapping of PARP1 on chromatin (*43*). We therefore asked whether non-covalent DNMT1 trapping could similarly trigger DNMT1 SUMOylation. To test this, we used GSK-3484862, a recently developed DNMT1 inhibitor that intercalates between two adjacent GC base pairs, inhibits DNMT1 enzymatic activity and induces DNA demethylation (*44*). The addition of GSK-3484862 to the egg extracts induced DNMT1 accumulation and its modification on chromatin, and also increased the recruitment of xDAXX (Fig. 5A). Importantly, this DNMT1 modification was suppressed by ML-792 (Fig. 5A). Consistently, immunoprecipitation of DNMT1 revealed SUMO2/3 conjugation in the presence of GSK-3484862 (Fig. 5B). Furthermore, xDAXX depletion markedly suppressed DNMT1 SUMOylation in response to GSK-3484862 treatment (Fig. 5C and D). Taken together, these results indicate that xDAXX also promotes the SUMOylation of non-covalently trapped DNMT1 on chromatin.

### DAXX inhibition enhances DAC sensitivity in THP-1 cells

**Fig. 6:**
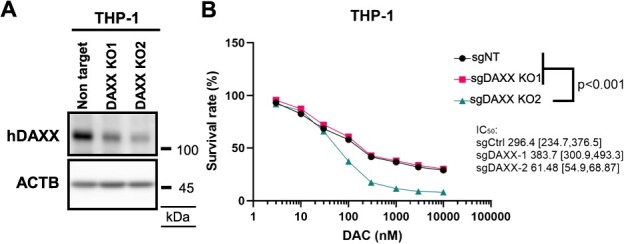
**DAXX inhibition enhances DAC sensitivity in THP-1 cells.** (A) Immunoblotting results showing the DAXX KO efficiency of THP-1 cell lysates using indicated antibodies. (B) Survival analysis results showing the IC_50_ of DAC in sgNT, sgDAXX-1 and sgDAXX-2 THP-1 cells. Cells were treated with DAC at the indicated concentration for 72 h (*n* = 6 for each group). IC_50_ and 95% confidence interval were calculated using a non-linear regression model and profile likelihood method.

DAC exhibits antitumour activity and is clinically approved for the treatment of MDS and AML (*45*). Recent work has clarified that the cytotoxic effect of DAC in myeloid malignancies is mediated through the formation of DNMT1-DNA crosslinks (*18*). Importantly, SUMOylation of DNMT1 is required for the subsequent ubiquitination and proteasomal degradation of the crosslinked protein (*14*, *22*, *24*). Therefore, we examined whether DAXX knockout influences the sensitivity of myeloid malignancies to DAC. To address this issue, we targeted DAXX in the decitabine-resistant AML cell line THP-1 (*43*). Using two independent sgRNAs targeting DAXX, we assessed cellular sensitivity to DAC. Among the resulting cell lines, sgDAXX-2 exhibited more efficient suppression of DAXX expression than control cells (sgNT) or sgDAXX-1 (Fig. 6A). Importantly, consistent with reduced DAXX expression, the sgDAXX-2 cells exhibited a markedly reduced IC_50_ for DAC from 296.4 to 61.5 nM (Fig. 6B). The maximum plasma concentration of DAC reaches approximately 300 nM in clinical use (*46*), which suggests that DAXX knockout effectively sensitized THP-1 to DAC. These results support the idea that DAXX promotes DNMT1 SUMOylation, thereby facilitating DNMT1-DPCs resolution and attenuating DAC-induced cell death.

## Discussion

Here, we established a cell-free system derived from interphase *Xenopus* egg extracts that recapitulates DNMT1 trapping and SUMO2/3 conjugation at DNMT1-DNA crosslinks. In interphase *Xenopus* egg extracts, SUMOylated DNMT1 did not undergo STUbL-dependent ubiquitination and proteasomal degradation, likely because RNF4 E3 ligase activity is stimulated by mitotic CDK (*47*, *48*). Using this system, we identified DAXX as a factor recruited to 5-aza-dCTP-treated chromatin that promotes DNMT1 SUMOylation. In agreement with this finding, DAXX knockout in the DAC-resistant AML line THP-1 increased DAC sensitivity, suggesting that DAXX-mediated DNMT1 SUMOylation is highly conserved in humans, contributing to cellular tolerance to DAC and facilitating resolution of DNMT1-DPCs.

Notably, it has been reported that a catalytically inactive DNMT1 mutant failed to undergo SUMOylation after DAC treatment (*18*, *22*), suggesting that covalent trapping is required for DNMT1 SUMOylation. Yet our findings showed that the non-covalent DNMT1 inhibitor GSK-3484862 elicited DAXX-dependent DNMT1 SUMOylation, in line with a previous report that GSK-3484862 targets DNMT1 for proteasomal degradation in mammalian cells (*49*). More broadly, non-covalent engagement with chromatin can trigger selective SUMOylation of many chromatin-bound proteins, promoting displacement or degradation, often via STUbL pathways (*25*, *48*). Consistent with this, GSK-3484862 caused robust DNMT1 accumulation on chromatin in *Xenopus* egg extracts. We therefore propose that DAXX-dependent SUMOylation limits DNMT1 trapping on chromatin under conditions of defective maintenance DNA methylation, even in the absence of covalent crosslink. Taken together, the results suggest that prolonged chromatin retention, rather than crosslink chemistry alone, can act as the proximal trigger for DNMT1 SUMOylation. Future work should test whether the catalytically inactive mutant accumulates on chromatin to a degree sufficient to elicit DAXX-dependent SUMOylation.

Mechanistically, our data suggest that DAXX functions as an elongator of SUMO2/3 chains on DNA-trapped DNMT1. In *Xenopus* egg extracts, DAXX accumulation on 5-aza-dCTP-treated chromatin was blocked by SUMO E1 inhibition and by DNMT1 depletion. These results indicate that DAXX loading depends on DNMT1 SUMOylation and are consistent with the impaired chromatin recruitment of DAXX SIM mutants. Prior studies show that DAXX, UBC9 and SUMO can assemble a ternary complex through a non-covalent UBC9-SUMO interaction (*38*), and that DAXX engages the SUMO E3 ligase PIAS1 via its C-terminal SIM (50). Taken together, these observations support a model in which DAXX acts as an adaptor linking SUMOylated DNMT1 to the UBC9-SUMO module or an E3 ligase such as PIAS1, locally enhancing SUMO conjugation on DNMT1. Besides, it would be possible that DNMT1 trapped on chromatin could increase local SUMO density and residence time, thereby facilitating DAXX recruitment and further SUMOylation. On the other hand, it has been mentioned that SUMO signalling recruits the DAXX-ATRX complex, functioning as a histone H3.3 chaperone, to chromatin to promote chromatin remodelling, as reported for SUMO-modified MORC3 and PRC1 (*40*, *41*, *50*, *51*). By analogy, SUMOylated DNMT1 at DNMT1-DNA crosslinks potentially provides a platform to recruit the DAXX-ATRX complex, thereby remodelling chromatin around the lesion and facilitating downstream processing. Investigating whether SUMOylated DNMT1 promotes the formation of such complexes at DPC sites will be an important direction for future studies.

## Materials and Methods

### Interphase egg extracts derived from *Xenopus* unfertilized eggs

*Xenopus laevis* were obtained from Kato-S Kagaku and handled according to the guidelines for animal experiments at the University of Tokyo. Interphase egg extracts were prepared as described previously (*35*). Briefly, unfertilized *X. laevis* eggs were dejellied in 2.5% thioglycolic acid–NaOH (pH 8.2) and washed three times with 1× Marc’s Modified Ringer Solution (MMR) (100 mM NaCl, 2 mM KCl, 1 mM MgCl_2_, 2 mM CaCl_2_, 0.1 mM EDTA and 5 mM HEPES–NaOH [pH 7.5]). Eggs were activated with 0.3 μg/ml calcium ionophore supplemented with 1× MMR and followed by washing in 0.2× MMR and then in 0.5× extraction buffer (EB) (50 mM KCl, 2.5 mM MgCl_2_, 10 mM HEPES–KOH [pH 7.5] and 50 mM sucrose). The eggs were packed into tubes by centrifugation (BECKMAN, Avanti J-E, JS-13.1 swinging rotor) for 1 min at 190 × *g*. Crude extracts were harvested by centrifugation for 20 min at 18,973 × *g*, and they were supplemented with 2 μg/ml aprotinin, 5 μg/ml leupeptin, 1 mM dithiothreitol (DTT), 50 μg/ml cycloheximide and 20 μg/ml cytochalasin B. Cytoplasmic extracts were then collected by ultracentrifugation (Hitachi, CP100NX, P55ST2 swinging rotor) for 20 min at 48,400 × *g*, frozen in liquid nitrogen and stored at −80 °C.

### Chromatin isolation

Interphase egg extracts were supplemented with an ATP regeneration system (ATP-RS, 20 mM phosphocreatine, 2 mM ATP, 5 μg/ml creatine phosphokinase). *Xenopus* sperm nuclei (3000–4000 nuclei/μl) were added to the extracts and incubated at 22°C. At the indicated time points, aliquots (15–20 μl) were diluted 10-fold in chromatin purification buffer (CPB) (50 mM KCl, 5 mM MgCl_2_ and 20 mM HEPES–KOH [pH 7.6]) containing 0.1% Nonidet P-40 (NP-40), 2% sucrose and 2 mM N-ethylmaleimide (NEM), and incubated for 5 min at 4°C. Diluted extracts were layered onto 1.5 ml of CPB containing 30% sucrose and centrifuged for 10 min at 15,000 × *g*. After the supernatants were removed, the pellets were collected as chromatin fraction, resuspended in 1× Laemmli sample buffer and boiled for 5 min at 100°C. These samples were analyzed by western blotting using antibodies against the indicated proteins.

For DNMT1 inhibition, egg extracts were incubated with 50 μM 5-aza-dCTP (Jena Bioscience) or with 100 μM GSK-3484862 (MedChemExpress). For the inhibition of the SUMOylation pathway, egg extracts were incubated with UBC9-dominant negative, which were kindly gifted from Dr Azuma (the University of Kansas) or with 50 μM ML-792 (MedChemExpress).

### Immunoprecipitation

For chromatin solubilization, 50 μl of egg extracts were supplemented with an ATP-RS and *Xenopus* sperm nuclei. After incubation at 22°C, the extracts were diluted 5-fold in CPB containing 0.1% NP-40, 2% sucrose and 2 mM NEM and incubated for 5 min at 4°C. The diluted extracts were then layered onto a sucrose cushion (490-μl CPB containing 30% sucrose layered over 10-μl CPB containing 2 M sucrose) and centrifuged for 10 min at 10,000 × g. The supernatant was aspirated, leaving 50 μl of solution. The solution was diluted with 50 μl of 2× benzonase solution (100 mM Tris–HCl [pH 7.5], 300 mM NaCl, 2% NP-40, 2 mM MgCl_2_ and 0.2% SDS) containing 4 U/μl Benzonase (MERCK), vortexed and incubated for 15 min at 37°C. After incubation, the solution was centrifuged for 10 min at 16,510 × *g*. The supernatant was collected as the insoluble chromatin fraction. Ten microliters of an aliquot was mixed with Laemmli sample buffer and boiled for 5 min at 100°C. The remaining samples were frozen in liquid nitrogen and stored at −80°C. For the immunoprecipitation, the chromatin fraction was diluted 10-fold in lysis buffer (150 mM NaCl, 1% Triton X-100, 1 mM EDTA, 15 mM Tris–HCl [pH 8.0]).

For the preparation of antibody-bound beads, 10 μl of protein A agarose (GE Healthcare) was incubated with 5 μl of antiserum overnight at 4°C. These beads were incubated with diluted chromatin fraction at 4°C for 2 h. After the incubation, these beads were washed with CPB containing 2% sucrose and 0.1% Triton X-100, resuspended in Laemmli sample buffer and boiled for 5 min at 100°C.

### Immunodepletion

Rabbit polyclonal antibodies raised against *Xenopus* DNMT1 (xDNMT1) have been described previously (*30*). *Xenopus* DAXX antibodies used for immunoblotting and immunoprecipitation were raised by Hokudo against a bacterially expressed recombinant protein fragment encoding 362–916 amino acids from xDAXX. For immunodepletion, 200 μl of antiserum was coupled to 60 μl of recombinant protein A-Sepharose (rPAS, GE Healthcare). Antibody beads were washed with PBS containing 0.05% NP-40 and then supplemented with 6 μl of fresh rPAS beads. The antibody beads were washed twice in CPB containing 2% sucrose, split into two portions and used to deplete 100 μl of extracts in two rounds at 4°C, each for 1 h. The antibody beads were separated from the egg extract by centrifugation for 1 min at 1000 × *g*. Mock depletion was performed using preimmune antiserum.

### Antibodies used for western blotting

The following rabbit polyclonal antibodies were used for Western blotting: xDNMT1 (1:500) (*30*); xUHRF1 (1:1000) (*30*); xPAF15 (1:500) (*32*); xDAXX (1:500; this study); SUMO2/3 (1:1000; Abcam, D7810); hDAXX (1:1000; MERCK, ab32140); and histone H3 (1:3000; Abcam, ab1791). Mouse monoclonal antibodies against the following proteins were used for Western blotting: FLAG (1: 1000, Sigma-Aldrich, F3165) and β-actin (1:1000; Santa Cruz Biotechnology, sc-69,879). The following secondary antibodies were used for detection: HRP-linked Anti-Mouse (1:3000, cytiva, NA931) and HRP-linked Anti-Rabbit (1:3000, cytiva, NA934).

### Chromatin mass spectrometry

Chromatin isolated from egg extracts was digested using the EasyPep Mini MS Sample Prep kit (Thermo Fisher Scientific) according to the manufacturer’s protocol. After egg extract was supplemented with ATP-RS and sperm nuclei and incubated at 22°C, chromatin was isolated using a sucrose cushion. Isolated chromatin was resuspended in 50 μl of lysis buffer containing 0.5 μl of universal nuclease and centrifuged for 10 min at 16,000 × *g*. The supernatant was collected as the soluble chromatin fraction. Ten micrograms of peptides from the soluble chromatin fraction were labelled with 100 μg of TMTpro mass tag (TMT) reagent (Thermo Fisher Scientific) according to the manufacturer’s protocol. After TMT labelling, the 11 sample channels were combined at equal ratios, dried using a SpeedVac and resuspended in 0.1% TFA. Samples were fractionated into eight fractions using High pH Reversed-Phase Peptide Fractionation Kit (Thermo Fisher Scientific) according to the manufacturer’s protocol. One microgram of peptides from each fraction was analyzed by LC–MS/MS using an EASY-nLC 1200-connected Orbitrap Fusion Lumos Tribrid mass spectrometer (Thermo Fisher Scientific) equipped with FAIMS-Pro ion mobility interface (Thermo Fisher Scientific). Peptides were separated on an analytical column (C18, 1.6-μm particle size × 75-μm diameter × 250-mm length, Ion Opticks) using 4-h gradients (0% to 28% acetonitrile over 240 min) at a constant flow of 300 nl/min. Peptide ionization was performed using Nanospray Flex Ion Source (Thermo Fisher Scientific). FAIMS-Pro was set to three phases (−40, −60 and − 80 CV), and a ‘1 sec cycle for a phase’ data-dependent acquisition method was applied where the most intense ions in every 1 sec were selected for MS/MS fragmentation by HCD. MS raw files were analyzed using the Sequest HT search programme within Proteome Discoverer 2.4 (Thermo Fisher Scientific). MS/MS spectra were searched against the SwissProt-reviewed mouse reference proteome (UniProt). TMTpro-based protein quantification was performed using the Reporter Ions Quantifier node in Proteome Discoverer 2.4. Exported data were visualized by Prism (https://www.graphpad.com/features).

### Cell culture

HEK293T cells were maintained in Dulbecco’s Modified Eagle’s Medium (DMEM high glucose, Nacalai Tesque) supplemented with 10% foetal bovine serum (FBS) and 1× penicillin/streptomycin/amphotericin B (Nacalai Tesque). THP-1 cells were maintained in RPMI-1640 medium (Nacalai Tesque) supplemented with 10% FBS and 1× penicillin/streptomycin/amphotericin B. Sf9 cells were maintained in ESF 921 insect cell culture medium (Funakoshi) supplemented with 10% FBS and 1× penicillin/streptomycin/amphotericin B. Cells were cultured at 37°C under 5% CO_2_ and normoxic conditions except that Sf9 cells were cultured at 27°C under 5% CO_2_.

### Plasmid construction

*Xenopus laevis* Daxx cDNA was amplified from an *X. laevis* cDNA egg library by PCR and ligated into the pTA2 vector. For protein expression in Sf9 cells, the 3× FLAG-tagged xDaxx gene was amplified by PCR and cloned into the pVL1392 vector. I7A/I8A or V911A/I912A point mutations were introduced in pVL1392-xDAXX using KOD-Plus Mutagenesis Kit (Toyobo). For xDAXX N-terminal/C-terminal deletion mutants, xDaxx cDNA was amplified by PCR and cloned into the pVL1392 vector using the In-Fusion system (Clontech).

For the establishment of DAXX-knockout THP-1 cells, gRNA pairs were annealed and ligated into BsmBI-digested lentiGuide-Puro (Addgene #52963) using T4 DNA ligase (TAKARA). These gRNA sequences were designed using CRISPick (https://portals.broadinstitute.org/gppx/crispick/public). The following gRNA sequences were used in this study: sgNT(non-target), 5′-CGCTTCCGCGGCCCGTTCAA-3′; sgDAXX-1, 5′-AATGTTGCAAGACAAAAGTG-3′; sgDAXX-2, 5’-CTGGCCTATAGTCATCTGTG-3′.

### Protein purification

Baculoviruses for overexpressing 3× FLAG-tagged xDAXX wild-type or mutants were generated using the BD BaculoGold Transfection Kit and the BestBac Transfection Kit (BD Biosciences) according to the manufacturer’s protocol. Sf9 insect cells were infected with the Baculovirus expressing various recombinant xDAXX proteins and incubated for 72 h at 27°C. Sf9 cell pellets were collected from a 3000-ml culture, resuspended with 45-ml lysis buffer (20 mM Tris–HCl [pH 8.0], 100 mM KCl, 5 mM MgCl_2_, 10% glycerol, 1% NP-40, 1 mM DTT, 5 μg/ml leupeptin, 2 μg/ml aprotinin, 20 μg/ml trypsin inhibitor and 100 μg/ml phenylmethylsulfonyl fluoride (PMSF)) and incubated on ice for 10 min. The soluble fraction was isolated by centrifugation for 15 min at 15,000 × *g* and incubated with 250-μl anti-FLAG M2 affinity resin (Sigma-Aldrich) equilibrated with a lysis buffer for 4 h at 4°C. The resin was separated from the soluble fraction by Muromac Mini Column (MUROMACHI CHEMICALS INC) and washed with wash buffer (20 mM Tris–HCl [pH 8.0], 100 mM KCl, 5 mM MgCl_2_, 10% glycerol, 0.1% NP-40, 1 mM DTT, 5 μg/ml leupeptin, 2 μg/ml aprotinin, 20 μg/ml trypsin inhibitor and 100 μg/ml phenylmethylsulfonyl fluoride (PMSF)) and elution buffer (EB) (20 mM HEPES–KOH [pH 7.5], 100 mM KCl and 5 mM MgCl_2_, and 1 mM DTT). 3× FLAG-tagged recombinant proteins were eluted in EB containing 1 mM DTT and 250 μg/ml 3× FLAG peptide. Eluted proteins were concentrated by using a Vivaspin 500 concentrator (SARTORIUS).

### The establishment of DAXX KO cell lines

Lentiviruses expressing Cas9 gene or the respective gRNAs were generated by co-transfecting to HEK293T cells with psPAX2 (Addgene #12260), pCMV-VSV-G (Addgene #8454) and lentiCas9-Blast (Addgene #52962) or the constructed lentiGuide-Puro containing the indicated gRNA by the calcium phosphate co-precipitation method.

After THP-1 cells were infected with the lentivirus for expressing Cas9 gene, cells were selected with 20 μg/ml blasticidin (Thermo Fisher Scientific) for 3 days. The selected cells expressing Cas9 were infected with the lentivirus expressing the gRNA. After selection with 1.25 μg/ml puromycin (Thermo Fisher Scientific), cell pellets were collected by centrifugation for 3 min at 1000 × *g*, resuspended in 1× Laemmli sample buffer and boiled for 5 min at 100°C.

### Cell viability assay

To evaluate the toxicity of decitabine, THP-1 cells were plated at a density of 1 × 10^4^ cells/well in 100 μl of RPMI1640 medium in 96-well plates. Cells were treated with the indicated concentrations of DAC for 72 h at 37°C. Ten microliters of Cell Counting Kit-8 (Dojindo) was added to each well and incubated for 1–2 h at 37°C. After incubation, the absorbance at 450 nm was measured using a CLARIOstar Plus microplate reader (BMG LABTECH).

### Statistical analysis

Dose–response curves and half-maximal inhibitory concentration (IC_50_) values were analyzed using non-linear regression in GraphPad Prism (version 8.4.3). A four-parameter logistic model with a variable Hill slope was fitted to the normalized cell viability data. To determine whether different cell lines shared a common IC_50_, an extra sum-of-squares *F* test was performed by comparing two models: one in which all datasets shared a single LogIC_50_ value, and another in which LogIC_50_ was independently estimated for each dataset. The 95% confidence intervals for parameter estimates were calculated using the profile likelihood method. All curve-fitting and statistical tests were conducted in GraphPad Prism.
